# Exposure Stress Induces Reversible Corneal Graft Opacity in Recipients With Herpes Simplex Virus-1 Infections

**DOI:** 10.1167/iovs.16-19673

**Published:** 2017-01

**Authors:** Alexander M. Rowe, Hongmin Yun, Robert L. Hendricks

**Affiliations:** 1 University of Pittsburgh Department of Ophthalmology, Pittsburgh, Pennsylvania, United States; 2 University of Pittsburgh Department of Immunology, and Microbiology and Molecular Genetics, Pittsburgh, Pennsylvania, United States

**Keywords:** corneal transplantation, herpes simplex keratitis, optic neuropathy

## Abstract

**Purpose:**

Most of the inflammation in murine herpes simplex virus type 1 (HSV-1)-induced stromal keratitis (HSK) is due to exposure stress resulting from loss of corneal nerves and blink reflex. Corneal grafts often fail when placed on corneal beds with a history of HSK. We asked if corneal exposure contributes to the severe pathology of corneal grafts on HSV-1–infected corneal beds.

**Methods:**

Herpes simplex virus type 1–infected corneas were tested for blink reflex. Opacity and vascularization were monitored in allogeneic and syngeneic corneal grafts that were transplanted to corneal beds with no blink reflex or to those that retained blink reflex in at least one quadrant following infection.

**Results:**

Retention of any level of blink reflex significantly reduced inflammation in HSV-1–infected corneas. Corneal allografts placed on HSV-1–infected beds lacking corneal blink reflex developed opacity faster and more frequently than those placed on infected beds that partially or completely retained blink reflex. Corneal grafts placed on infected corneal beds with no blink reflex rapidly became opaque to a level that would be considered rejection. However, protecting these grafts from exposure by tarsorrhaphy prevented or reversed the opacity in both syngeneic and allogenic grafts.

**Conclusions:**

Exposure due to HSV-1–engendered hypoesthesia causes rapid, severe, persistent, but reversible opacification of both allogeneic and syngeneic corneal grafts. This opacity should not be interpreted as immunologic rejection. Exposure stress may contribute to the high rate of corneal graft pathology in patients with recurrent HSK.

Corneal transplantation has long been the most commonly performed transplant surgery in the United States and Great Britain.^1,2^ Mechanisms of immunologic corneal transplant rejection have been largely defined in rodent models, particularly mice.^3–8^ Graft opacity and vascularization served as the main criteria for graft rejection in most of these studies. In a 2002 perspective paper,^9^ the authors state that graft rejection in the mouse and rat models can be compared by simply grading the graft opacity level. However, the authors qualify the statement with the caveat that transient opacification of corneal grafts referred to as “rejection episodes” can occur and limit the reliability of graft opacity as the sole criterion for rejection.^9^ Although it was demonstrated that changing surgical techniques could reduce the incidence of these rejection episodes, the conclusion was that no good explanation for this phenomenon was available. Thus, there is a need for a more thorough understanding of the mechanisms of graft opacity in the mouse corneal transplantation model.

Here, we use corneal herpes simplex virus-1 (HSV-1) infections to create a physiologically relevant high-risk model of corneal graft rejection. Corneal HSV-1 infections are both a major reason for corneal transplants, and a major cause of corneal transplant rejection.^10^ Herpes simplex virus-1 corneal infections result in leukocytic infiltration that can be subclinical or manifest as herpes stromal keratitis (HSK), an immunopathological process that represents the leading infectious cause of blindness in the United States.^11^ During primary HSV-1 infections, the virus establishes a latent infection in sensory ganglia that upon reactivation can serve as a source of virus for recurrent bouts of HSK. Thus, one could hypothesize that the high rate of sustained corneal graft opacity on HSV-1–infected corneal beds might result from: (1) residual inflammation and vascularization in the recipient cornea at the time of surgery, and/or (2) reactivation of latent virus and shedding at the corneal surface resulting in direct viral cytopathic damage or induction of damaging inflammation in the corneal graft. Indeed, evidence in support of both of these mechanisms has been reported.^12–17^

An association of HSK and other viral infections of the cornea with a loss of corneal sensation and exposure stress is well-established in humans.^18–21^ Recent findings demonstrate that the HSV-1–induced loss of corneal sensation is associated with partial or complete loss of corneal sensory nerves,^21–23^ and manifests as a loss of blink reflex and corneal exposure and desiccation.^22^ Indeed, it was shown that most of the inflammation associated with HSK resulted from corneal exposure and could be largely prevented or resolved by performing a protective tarsorrhaphy.^22^ Interestingly, mice developed severe HSK subsequent to a complete loss of corneal blink reflex.^22^ Although corneal grafts inherently lack sensation due to severing of corneal nerves during excision, sensation is normally retained in the corneal bed. In contrast, when placed on an HSV-1–infected bed both the corneal graft and the surrounding corneal bed might lack sensation leading to exposure-induced inflammation in the corneal graft. These findings led us to hypothesize that HSV-1–induced corneal exposure might constitute an additional cause for the development of sustained opacity of corneal grafts placed on HSV-1–infected corneal beds. Our findings support this hypothesis. Furthermore, we demonstrate that in order to correlate persistent opacity with graft rejection, one must consider a contribution of long-term exposure stress to graft opacity.

## Methods

### Animals

Female BALB/c and C57BL/6 mice purchased from Jackson Laboratories (Bar Harbor, ME, USA) were housed in the Animal Resource Facility at the University of Pittsburgh Medical Center (Pittsburgh, PA, USA). The use of animals was in accordance with the ARVO Statement for the Use of Animals in Ophthalmic and Vision Research, and all procedures were approved by the University of Pittsburgh Institutional Animal Care and Use committee.

### Viral Infection

The scarified corneas of female 6- to 12-week-old BALB/c mice were infected by topically applying 1 × 10^4^ plaque forming units (pfu) of HSV-1 strain KOS. Briefly, the mice were anesthetized with a mixture of ketamine and xylazine (Henry Schein Animal Health, Dublin, OH, USA) at doses 150 and 14 mg/kg of body weight, respectively. Anesthesia was delivered to the mice via intraperitoneal injection using a 30-G needle. Mice then received unilateral corneal scarification and topical virus application. The scratches on the corneal epithelium were deep enough to reach but not penetrate the basement membrane.

### Corneal Transplantation and Tarsorrhaphy

Tarsorrhaphy was performed under anesthesia using a surgical microscope (Olympus SZX16; Olympus Life Science, Tokyo, Japan). For infected but nontransplanted mice the procedure was performed after the skin disease and belapheratitis resulting from HSV-1 infection had resolved (generally around 15 days postinfection [dpi]). Three mattress stitches were made with 8-0 silk suture (8-0 coated vicryl, polyglactin 910 suture [TG140-6; 6.5 mm, 3/8 circle]; Cincinnati, OH, USS) through the interstitial part of the eyelids after both edges of the eyelids were mildly cut with scissors. The adhesion of the eyelids prevented the mice from opening their eyes, protecting the corneal surface from exposure, and desiccation following the loss of corneal sensation. Tarsorrhaphy was also performed directly after some transplantations and maintained for periods of time dictated by the individual experiments.

Allogeneic and syngeneic orthotopic corneal transplantations were performed on BALB/c mice. Donor corneas (2-mm diameter) from naïve C57BL/6 or BALB/c mice were excised and transplanted into the recipient cornea graft bed. The cornea graft was secured with eight interrupted 11-0 nylon sutures (Sharpoint, Nylon black monofilament, 5"/13cm, 0.1 metric, 11-0; Surgical Specialties Corporation, Reading, PA, USA).

### Scoring of Corneal Opacity, Assessment of Corneal Hypoesthesia, and Quantification of Neovascularization

For corneas without tarsorrhaphy, corneal opacities were scored at least twice a week in a masked fashion by two investigators with a high degree of consensus. Opacification was scored based on the following parameters: 0 = clear graft, 0.5 = small isolated imperfections or regions of translucence in the graft, 1 = minimal translucence or haziness extending over the entire graft with complete visualization of the iris and pupil, 1.5 = translucence or haziness extending over the entire graft and/or regional opacity in a minority of the graft, 2 = stromal opacity over the majority of the graft with visualization of the pupil margin and the iris structures, 2.5 = stromal opacity extending over the majority of the graft with only small regional translucence allowing very limited and strongly obscured visualization of the iris, 3 = complete stromal opacity with no visualization of the iris structures, 3.5 = complete stromal opacity associated with associated pathology such a granuloma or ulcer in the graft, 4 = destruction of the graft, a loss of graft integrity or graft perforation. Opacity associated with HSV-1 infections was assessed using the same scale and method used for measuring sustained graft opacity, but considered the whole cornea rather than just the graft. For the survival curves a graft was recorded as opaque when two consecutive scores of 3 or greater were observed. The incidence of sustained opacity was then recorded as the date of the first opacity score of 3 or greater. Measurement of neovascularization included the entire cornea, not just the graft, and the same scoring system was used for both transplanted and nontransplanted corneas. Briefly, the cornea was visually divided into four quadrants, and each quadrant was scored as 0 = no vessels visible, 1= vessels extend into the corneal bed, 2 = vessels extending up to graft interface or 50% of the distance to the center of the cornea, 3 = vessels extending into the graft but not reaching the center of the graft or extend 50% to 75% of the distance to the graft center, and 4 = vessels extending to the central cornea. The scores for all four quadrants were added to achieve a final score on a scale 0 to 16.

The corneal blink reflex was tested and recorded as positive or negative by loosely holding the mouse and touching four areas in the periphery of the cornea and the center of the cornea with a sterile plastic probe being careful to avoid touching the eyelashes and whiskers (see Supplementary Videos S1, S2). A loss of blink reflex denoted a complete loss of corneal sensation such that the mouse failed to blink when any area of the cornea was touched. A positive score indicated retention of some degree of sensation such that the mouse blinked when at least one area of the cornea was touched.

### Assessment of Viral Copy Number in the Trigeminal Ganglion (TG)

Groups of mice received HSV-1 corneal infections and TGs were excised following the resolution of skin disease and blepharitis (>15 dpi). The TGs were placed into a DNA preserving tissue digestion buffer purchased from Qiagen (Qiagen, Germantown, MD, USA). The tissue was digested in the presence proteinase K and the DNA was isolated using Qiagen DNAeasy kit as per manufactures instruction. The viral copy number was assessed by using a real-time PCR protocol employing a labelled probe (5′-(FAM)TCCGGACCACTTTTC(NFQ)-3′), which recognizes the gene for glycoprotein H (gH). The reaction was carried out using TaqMan Universal PCR Master Mix (Thermo Fisher Scientific, Waltham, MA, USA). Because HSV-1 contains a single copy of gH, we compared the Ct values with that of a plasmid standard and then used a liner regression to calculate copy number. Mathematical and statistical analysis was performed using Applied Biosystems StepOne software (Thermo Fisher Scientific, Waltham, MA, USA) and GraphPad Prism Software (GraphPad Software, Inc., La Jolla, CA, USA).

### ELISA

Splenocytes from C57BL/6 or BALB/c mice were exposed to 3000 rad of gamma radiation and used as stimulator cells. The graft recipient splenocytes were then incubated in triplicate cultures with an equal number of C57BL/6 stimulator cells (experimental group), BALB/c stimulator cells (negative control), or BALB/c stimulator cells plus 0.1 μg/mL of anti-CD3 (Clone 145-2C11; BD Pharmigen, Franklin Lakes, NJ, USA) as a positive control. After 3 days in culture, the supernatants and the cells were harvested.

The supernatants were analyzed by sandwich ELISA for IFN-γ. Briefly, Immulon 4HBX high binding polystyrene ELSIA plates (Thermo-Fischer, Rochester, NY, USA) were coated with anti–IFN-γ (clone R4-6A2) over night at 4°C, washed with PBS + 0.05% tween 20 (Sigma-Aldrich Corp., St. Louis, MO, USA), and the wells were blocked for 1 hour with PBS + 0.05% tween and 1% BSA (Sigma-Aldrich Corp.). The wells were washed and then loaded with either 100 μL of either diluted supernatant from the lymphocyte cultures, or of various concentrations of a purified mouse IFN-γ standard. The plates were incubated overnight at 4°C, the wells were washed and loaded with biotin conjugated anti–IFN-γ (clone XMG1.2; BD Pharmigen). After 1 hour at room temperature the wells were washed, loaded with streptavidin conjugated to horseradish peroxidase, and incubated for 30 minutes at room temperature. The wells were washed and loaded with 100-μL TMB peroxidase substrate (Life Technologies, Fredrick, MD, USA). Color development was stopped by addition of 50 μL of 1N H_2_S0_4_, and absorbance was read at 450 nm using a Synergy 2 plate reader (BioTek, Winooski, VT, USA).

## Results

### Severe Opacity and Vascularization in HSV-1–Infected Corneas is Closely Associated With Loss of Corneal Sensitivity

**Figure 1:**
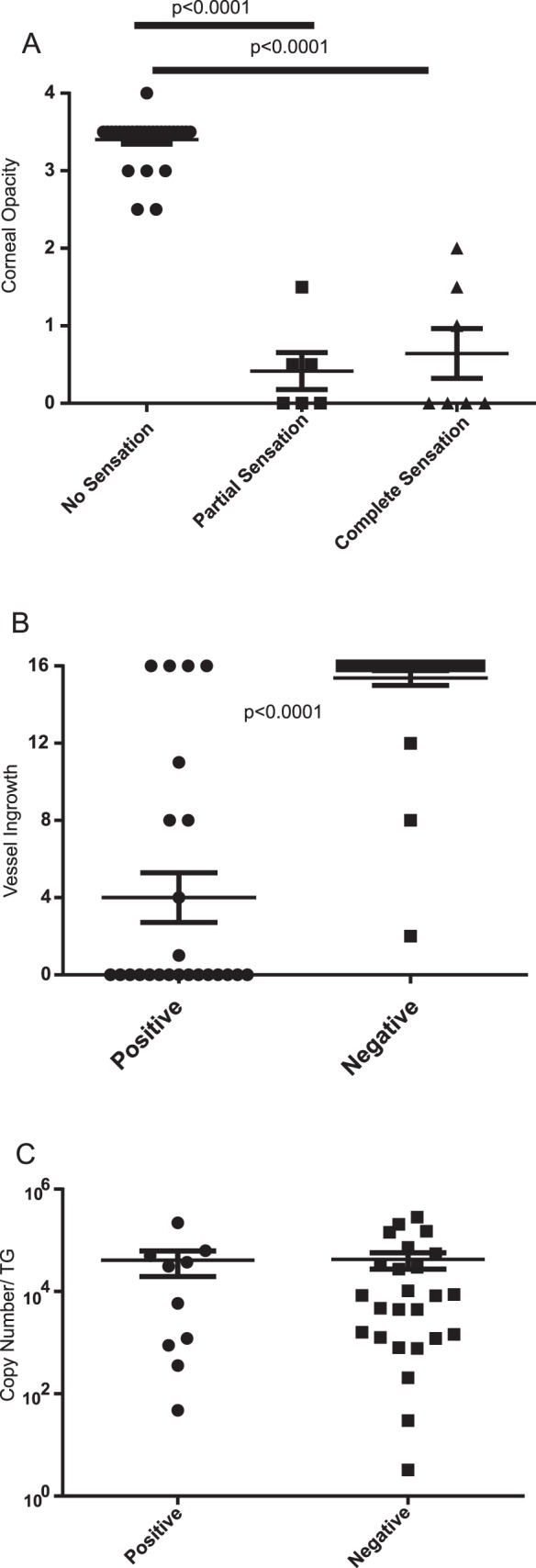
Herpes stromal keratitis develops only when corneal blink reflex is completely lost. The corneas of BALB/c mice were infected with 1 × 10^4^ pfu of HSV-1. Once blepharitis resolved so corneas could be examined (∼15 dpi) blink reflex was recorded while touching all four quadrants of the peripheral cornea and the central cornea. The corneas were also scored for opacity on a 4-point scale and for neovascularization on a 16-point scale as described in Methods. (**A**) Opacity scores of infected corneas that lost blink reflex in all areas of the cornea (no sensation), lost blink reflex in some but not all areas of the cornea (partial sensation), or retained blink reflex in all areas of the cornea (complete sensation). Mean opacities were compared statistically via a 1-way ANOVA followed by Tukey posttest and *P* values of significant differences are shown. (**B**, **C**) Mice were assigned to groups that retained blink reflex in some or all areas of the cornea (positive) or lacked blink reflex in all areas of the cornea (negative). (**B**) Blood vessel ingrowth was scored for positive and negative corneas. (**C**) Trigeminal ganglia were excised from mice that were positive or negative for blink reflex and viral genome copy number was quantified by real-time quantitative PCR as described in Methods. Mean and SEM are denoted by *horizontal* and *vertical lines*, respectively. The significance of group differences in vascularization scores and viral copy number was determined via a Mann-Whitney rank sum test. Data were pooled from two (**A**, **B**) or four experiments (**C**).

Corneal infection of BALB/c mice with an infectious dose of 1 × 10^5^ pfu of HSV-1 KOS induces severe vascularization, opacity, and complete loss of corneal nerves and corneal sensitivity as assessed by blink reflex in nearly 100% of mice.^22^ A lower dose of the same virus was shown to induce severe HSK in only 50% of mice, but the relationship between loss of corneal sensitivity and development of HSK was not investigated in that study.^24^ Therefore, BALB/c mice received corneal infections with a suboptimal (1 × 10^4^ pfu) dose of HSV-1 KOS. At 15 to 28 days post infection (dpi) corneas were tested for blink reflex by touching four quadrants of the peripheral cornea and the central cornea (illustrated in Supplementary Videos S1, S2), and for opacity and vascularity by microscopic examination. This dose of HSV-1 resulted in complete loss of blink reflex in 65% of the mice, and those mice all developed severe corneal opacity (Fig. 1A). Corneal opacity and vascularity were significantly less severe in corneas that partially or fully retained corneal blink reflex (Figs. 1A, 1B). Groups of mice that fully retained blink reflex in all areas of the infected cornea developed similar opacity to those that retained blink reflex in only 1 to 4 areas of the infected cornea (Fig. 1C). Loss of corneal blink reflex results from retraction of nerve fibers derived from the TG. However, there was no significant difference in the viral genome copy number in TG of mice that fully lost blink reflex when compared with those that partially or fully retained corneal blink reflex (Fig. 1D).

### Loss of Corneal Sensation in HSV-1–Infected Graft Beds is Associated With Rapid Development of Severe Opacity in Corneal Allografts

**Figure 2:**
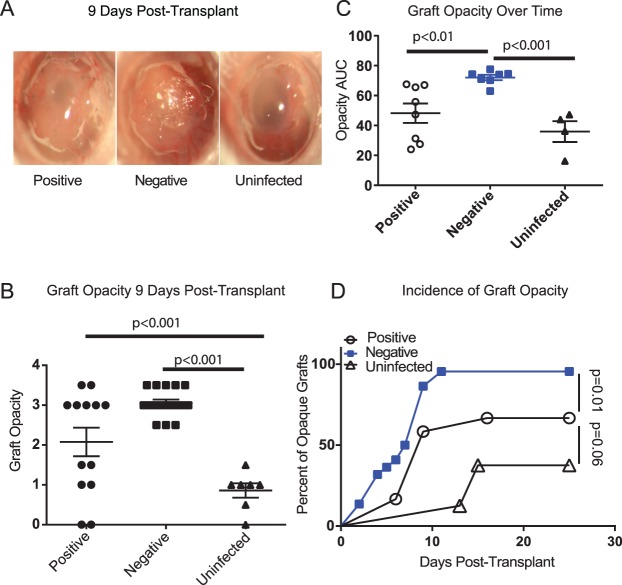
Rapid opacification of corneal allografts in recipients lacking corneal sensation. Corneas of BALB/c mice were mock infected or infected with 1 × 10^4^ pfu of HSV-1. Once blepharitis resolved so corneas could be examined (>15 dpi) infected corneas were tested for blink reflex by touching all four quadrants of the peripheral cornea and the central cornea. Allogeneic donor C57BL/6 corneas were then grafted to noninfected recipient corneal beds, or infected recipient corneal beds that either completely lost blink reflex (negative), or retained blink reflex when one or more areas of the cornea were touched (positive). (**A**) Representative images of corneal grafts were obtained 9 days after transplantation. Note the reduced opacity and vascularization in corneal grafts that were placed on positive or uninfected corneal beds relative to those placed on negative corneal beds. (**B**) Opacity scores of corneal grafts recorded at 9 days post transplant. (**C**) The opacity of grafts was scored at least twice a week for 28 days, plotted, and total opacity of each cornea was determined as the area under the curve (AUC). (**D**) Graft opacity was recorded and grafts considered “opaque” when they achieved and maintained an opacity score greater than or equal to 3, used in many studies to denote graft rejection. Data are presented as the percent of corneal grafts that were opaque at various days post transplant. The mean ± SEM are presented as *horizontal* and *vertical lines*, respectively for (**B**, **C**). The statistical significance of group differences was determined using a 1-way ANOVA with Tukey's posttests (**B**, **C**), or a Log-rank (Mantel-Cox) survival test (**D**).

Corneal allografts (C57BL/6 donors to BALB/c recipients) were placed on HSV-1–infected corneal beds that completely lost blink reflex or maintained some level of blink reflex, or on noninfected corneal beds. Graft opacity was measured 9 days post transplant and representative photographs are provided (Fig. 2A). Fourteen of 17 grafts placed on HSV-1–infected corneal beds lacking blink reflex exhibited greater than or equal to 3 opacity scores by 9 days post transplant, whereas only 7 of 13 grafts placed on infected corneal beds that retained some blink reflex, and none of the grafts placed on noninfected corneal beds achieved greater than or equal to 3 opacity scores at that time (Fig. 2B). The opacity score for each graft was plotted over a 28-day follow-up and total opacity expressed as the area under the curve (Fig. 2C). Grafts placed on HSV-1–infected corneal beds exhibited greater opacity when the beds completely lost blink reflex than when they maintained some level of sensitivity (Fig. 2C).

A sustained opacity score greater than or equal to 3 is a frequently used metric for defining immunologic rejection in mice. When placed on HSV-1–infected corneal graft beds that completely lost blink reflex, 95% of corneal allografts achieved a sustained opacity score greater than or equal to 3 within 11 days (Fig. 2D). In contrast, when placed on noninfected corneas only 38% of corneal allografts exhibited a sustained score greater than or equal to 3, and this frequency was not achieved until 15 days after graft placement. Corneal allografts that were placed on infected corneal beds that retained some level of blink reflex exhibited an intermediate level of pathology, with 67% achieving an opacity score greater than or equal to 3 within 15 days of placement. Sustained opacity developed more slowly in corneal grafts placed on HSV-1–infected corneas that retained some blink reflex relative to those that completely lost blind reflex, but more rapidly than those placed on noninfected corneas.

### Corneal Grafts Develop Severe, But Reversible Opacity When Placed on HSV-1–Infected Corneal Beds That Completely Lack Blink Reflex

**Figure 3:**
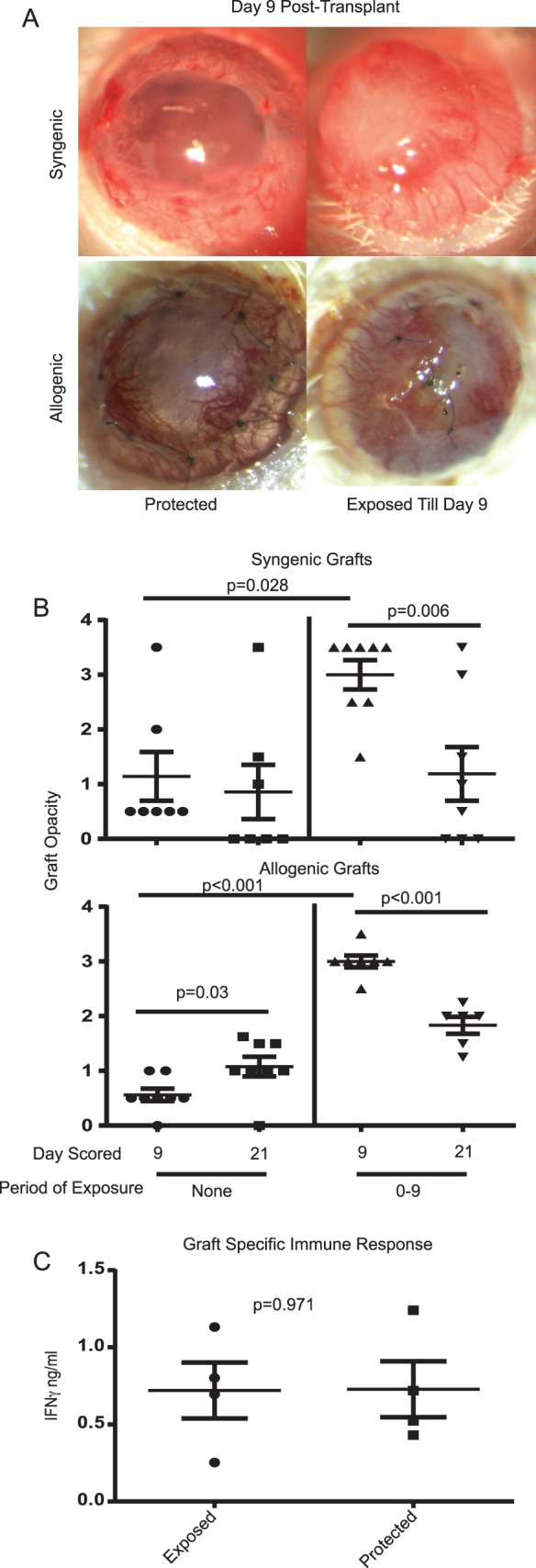
Opacity of syngeneic and allogeneic corneal grafts on HSV-1–infected beds lacking sensitivity is induced by exposure and reversible by tarsorrhaphy. Corneas of BALB/c mice were infected with 1 × 10^4^ pfu of HSV-1. At 28 days post infection, infected corneas that completely lost blink reflex in all areas of the cornea served as recipients of syngeneic BALB/c or allogenic C57BL/6 corneal grafts. Some of the grafts were protected from exposure by suturing the eyelids closed (tarsorrhaphy). (**A**) Representative images obtained 9 days post transplant show dramatically reduced opacity when syngeneic and allogeneic corneal grafts were protected from exposure by tarsorrhaphy. (**B**) Opacity scores were recorded at 9 and 21 days post transplant for corneal syngeneic or allogeneic grafts that were protected from exposure by tarsorrhaphy for the entire 21 day observation period (*left panel*) or were exposed for the first 9 days post transplant and then protected from 9 to 21 days post transplant (*right panel*). The significance of group differences was determined with a Student's *t*-test. (**C**) Spleens were harvested 21 days post transplant and cultured for 72 hours in the presence of irradiated C57BL/6 splenocytes. At 72 hours the amount of IFN-γ in the supernatant was assessed via ELISA. (**C**) The mean ± SEM values are presented as *horizontal* and *vertical lines*, respectively, and group means were compared via unpaired Student's *t*-test. Experiments presented were repeated with similar results.

Herpes simplex virus type 1 induced loss of corneal sensory nerves and blink reflex and the associated corneal exposure stress results in severe opacity and inflammatory infiltration in HSV-1–infected corneas.^22^ This inflammation is largely reversible by protecting the corneal surface with tarsorrhaphy.^22^ We hypothesized that exposure contributes to the severe opacity in allografts that are placed on corneal beds that have lost blink reflex. To investigate the contribution of exposure to graft opacity we placed corneal allografts (C57BL/6 donor to BALB/c recipient) or syngeneic grafts (BALB/c mice donors to BALB/c recipients) on HSV-1–infected corneas that completely lost blink reflex and had completely vascularized corneas. The mice were then divided into two groups. Tarsorrhaphy was performed on one group to prevent graft exposure. Graft opacity for the first group was measured at 9 days post transplant (Figs. 3A, 3B). The second group was left exposed during the first 9 days after transplant (Figs. 3A, 3B). Then both groups received a tarsorrhaphy that was maintained from day 9 until day 21 post transplant. When corneal grafts placed on infected beds lacking blink reflex were left exposed for 9 days both syngeneic and allogeneic corneal grafts achieved a mean sustained opacity score of greater than or equal to 3 (Figs. 3A, 3B). However, when these grafts were then protected from exposure by tarsorrhaphy from 9 to 21 days post transplant, opacity scores of both syngeneic and allogeneic grafts were significantly reduced (Fig. 3B). Herpes simplex virus type 1 in the tears or corneal buttons of recipient mice was undetectable by viral plaque assay, and HSV-1 genome levels in the tear film were at or below the level of quantification and did not significantly increase following transplantation (data not shown). These results suggest that graft opacity was not caused by release of the virus into the graft bed. These findings emphasize the tenuous nature of using an opacity score of greater than or equal to 3 to define graft rejection without controlling for sources of exposure stress.

Syngeneic grafts and allografts that were protected from exposure through the entire 21-day follow-up exhibited only mild opacity at 9 and 21 days post transplant (Figs. 3A, 3B). The mean opacity score of protected syngeneic grafts declined and most were completely clear by 21 days post transplant, consistent with surgical trauma as the cause of opacity. Opacity in protected allografts increased from 9 to 21 days but remained well below the level of opacity seen in unprotected corneas. These findings are consistent with the notion that an alloantigen response develops in corneal grafts that are placed on infected beds lacking sensitivity, but is masked by a severe inflammatory response resulting from graft exposure. In support of this notion, we show that spleen cells from allograft recipients lacking corneal sensitivity exhibit alloreactivity that is not eliminated by preventing exposure stress through tarsorrhaphy (Fig. 3C).

## Discussion

The mouse has been the primary animal model for researching the immunologic principles of corneal graft rejection.^8,25^ It has been argued, that in the mouse model measurements of corneal opacity are sufficient to determine if a cornea has undergone CD4^+^ T cell–mediated immunologic rejection.^9^ In many cases, this is a fair interpretation and depletion or manipulation of the CD4 T-cell response can prevent the onset of corneal graft opacity.^3,5–7,26^ Yet, this equation of corneal graft opacity with direct and permanent rejection of corneal tissue ignores other potential drivers of sustained graft opacity. Many studies, including some of our own, have not controlled for other causes of corneal opacity, such as exposure stress and neuropathy. We believe that for the mouse model to be an effective tool for researching corneal graft rejection the studies need to control as much as possible for factors such as exposure stress. In this study, we examined the fate of syngeneic and allogeneic grafts transplanted onto HSV-1–infected corneal beds. Herpes simplex virus type 1–infected BALB/c mice loose corneal blink reflex and develop a pathology that is at least in part due to the resulting exposure stress.^22^ Indeed, when infected mice lacking corneal sensitivity are depleted of CD4^+^ T cells or subjected to superior cervical ganglionectomy opacity resolves in conjunction with recovery of corneal sensory nerves and corneal sensitivity, suggesting that recovery of sensory nerve function is sufficient to restore clarity in infected corneas.^22,27^ Using this model, we have demonstrated that exposure stress can induce corneal opacity that mimics rejection and that this phenotype is reversible. That nerve damage and exposure keratitis is a driver of pathology in HSV-1–infected mouse corneas was only recently established.^22,23,27^ The results reported in this paper build upon and refine some of our previous finding regarding the nature of HSV-1 corneal infections and their contribution to graft rejection.

First, our earlier work demonstrated that induction of HSK is dose-dependent in BALB/c mice, with 100% disease incidence at an infectious dose of 1 × 10^5^ pfu of HSV-1 KOS, but only a 50% disease incidence at an infectious dose of 1 × 10^3^ pfu of the same virus.^24^ However, that study did not address the role of corneal hypoesthesia on HSK development. In our current study, we infected BALB/c mice with a suboptimal dose of HSV-1 and examined the association of pathology with loss of corneal sensation. At that infectious dose, mice exhibited either partial or complete loss of corneal sensation as measured by blink reflex. We observed a significant association between complete loss of corneal sensation (loss of blink reflex in all four quadrants of the peripheral cornea and the central cornea) and severe inflammation as measured by corneal opacity. In contrast, partial loss of corneal sensation (retention of blink reflex in one or more sectors of the cornea) was not associated with increased corneal inflammation (Fig. 1). Our previous study demonstrated that the inflammation in infected corneas that exhibit complete loss of sensitivity can be reversed by protecting the cornea from exposure suggesting that the severe inflammation reflects an exposure keratopathy resulting from the lack of blink reflex.^22^ Taken together these findings suggest that retention of sensitivity in any area of the cornea is sufficient to prevent development of severe exposure keratopathy. In agreement with this conclusion is our observation that infected corneas that completely lack sensitivity exhibit severe desiccation that is not observed in infected corneas that exhibit only partial loss of sensitivity (our unpublished observation).

Herpes simplex virus type 1 corneal infection results in rapid infiltration of leukocytes into the cornea and also viral invasion of sensory nerves and transmission to the TG. The pathology seen in mice resembles neurotropic keratitis, which can be caused by damage to TG.^28,29^ It is unclear if the corneal nerve degeneration is caused by inflammation at nerve termini, virus infection of the nerves, or a combination of both. Our observation that mice that completely lose cornea sensitivity have similar mean TG genome copy number to those with partial sensory loss is consistent with an inflammatory rather than a viral etiology (Fig. 1).

Our previous findings reported that both syngeneic and allogeneic corneal grafts rapidly developed opacity when placed on HSV-1–infected corneal beds that exhibit severe HSK-associated inflammation.^30^ Because both syngeneic and allogeneic grafts rapidly developed severe and sustained opacity, we concluded in that work that the inflammation resulted from an innate immune response emanating from the inflamed recipient corneal bed. Here, we have amended those findings. There is innate inflammation driving the rapid opacification of cornea grafts, but the driver of that inflammation is exposures stress. Thus, mice that retain blink reflex develop less severe opacity during the first 9 days post transplant (Fig. 2), and protecting the grafts from exposure stress prevents rapid graft opacification (Fig. 3). Perhaps most importantly, we found that exposure stress appears to mask the pathology associated with allogenic transplants. This tentative conclusion is based on the observation that when protected from exposure opacity of syngeneic grafts decreased from 9 to 21 days post transplant, whereas opacity of allografts progressed during the same period. These findings are consistent with the notion that the relatively mild and transient opacity seen in protected syngeneic grafts is the result of surgical trauma, whereas the progressive opacity in allografts reflects an ongoing alloresponse. Indeed, we show that these mice develop an alloresponse in their spleen that is not affected by tarsorrhaphy. It is possible that residual inflammation in HSV-1–infected corneal beds results in an enhanced alloresponse. However, enhanced alloreactivity does not appear to be a major factor contributing to graft opacity because opacity develops very rapidly (within 2 days) in both syngeneic and allogeneic grafts placed on HSV-1–infected beds.

Our study demonstrates that the enhanced sustained opacity in corneas transplanted to HSV-1–infected corneal beds can be reversed by tarsorrhaphy, a standard surgical procedure performed in animals and humans to prevent corneal exposure.^31^ The tarsorrhaphy does more than just protect from exposure; it also stops tear film drainage.^32^ Thus, the dramatic recovery we see in mice the received a tarsorrhaphy may be due to prolonged contact with components of tear film and lid mucosa as well the removal of the exposure stress.

Corneal transplantation eliminates sensitivity in the transplanted corneal button. However, our data emphasize the importance of retaining sensitivity in the surrounding corneal bed. If the corneal bed completely lacks sensitivity, the transplanted cornea will become rapidly opaque due to exposure keratopathy. This keratopathy is largely avoided if sensitivity is retained in even a single quadrant of the corneal bed or if the corneal bed is protected by tarsorrhaphy following transplant. Thus, assessments of sensitivity in the corneal bed should be included in any transplant model. In humans, consensual blink reflex might mitigate some or all of the impact of grafting to a graft bed that lacks sensation, but a possible contribution should be considered.

## Supplementary Material

Supplement 1

Supplement 2

Supplement 3
