# Supplementary material for: Exposure Stress Induces Reversible Corneal Graft Opacity in Recipients With Herpes Simplex Virus-1 Infections
Source: Invest Ophthalmol Vis Sci. 2017 Jan;58(1):35–41. doi: 10.1167/iovs.16-19673 (PMC5225994; doi:10.1167/iovs.16-19673)
Supplement: Supplement 1 [file iovs-57-15-03_s01.pdf]

Supplemental Movies: BALB/c mice infected with $1x10^4$ pfu of HSV-1 strain KOS were assessed for blink reflex at >28 dpi. The corneas of mice were touched in all four quadrants of the peripheral cornea and the central cornea with a sterile plastic probe. Mice that did not blink in response to touch received a score of 0 (S1). Infected mice that blinked when touched in all five location received a score of 5 (S2).
